# Similarities and differences in the prevalence and risk factors of suicidal behavior between caregivers and people with dementia: a systematic review

**DOI:** 10.1186/s12877-024-04753-4

**Published:** 2024-03-14

**Authors:** Mohd Afifuddin Mohamad, Mohammad Farris Iman Leong Bin Abdullah, Nurul Izzah Shari

**Affiliations:** 1 Department of Community Health, Advanced Medical and Dental Institute, Universiti Sains Malaysia, Kepala Batas, Pulau Pinang 13200 Malaysia; 2 School of Human Resource Development and Psychology, Faculty of Social Sciences and Humanities (FSSH), Universiti Teknologi Malaysia, Skudai, Johor 81310 Malaysia

**Keywords:** Suicidal behavior, People with dementia, Caregivers of people with dementia, Systematic review

## Abstract

**Background:**

People with dementia and their caregivers are prone to suicidal behaviors due to difficulty adjusting to their initial caregiving role and due to emotional disturbances resulting from deterioration of functioning. The present systematic review (1) explored the prevalence of and risk factors for suicidal behavior and (2) assessed the similarities and differences in the prevalence and risk factors for suicidal behavior between people with dementia and their caregivers.

**Methods:**

A comprehensive literature search for research articles published between 1950 and 2023 was carried out using major databases, such as Google Scholar, Web of Science, PubMed, Scopus, PsycINFO, EMBASE, the Cochrane Library, and Medline.

**Results:**

A total of 40 research articles were selected for review. A total of 12 research articles revealed that the prevalence of suicidal behavior among caregivers ranged from 4.7% to 26%. However, the risk of suicidal behavior among people with dementia was inconsistent, as only 17 out of 28 selected studies reported the risk of suicidal behavior among people with dementia. The risk factors associated with suicidal behavior among caregivers of people with dementia could be both self-related and care receiver-related factors, whereas risk factors in people with dementia were self-related factors. Notably, greater cognitive decline, which impairs individuals’ ability to carry out complex acts and planning, may lower their suicidal risk. Finally, assessment of the risk of bias indicated that 95% of the selected studies had unclear risk.

**Conclusion:**

Self-related and care receiver-related factors should be assessed among caregivers of people with dementia to evaluate the risk of suicidal behavior. In addition, we recommend evaluating suicidal risk in people with dementia in the early phase of dementia when cognitive decline is less severe. However, as the majority of the selected studies had unclear risk of bias, future studies with improved methodologies are warranted to confirm our study findings.

**Supplementary Information:**

The online version contains supplementary material available at 10.1186/s12877-024-04753-4.

## Introduction

Suicide is defined as any act of taking one’s own life on purpose [1]. Annually, suicide results in more than 700,000 life losses globally, and it is prevalent not only in developed countries but also in low- and middle-income countries [2]. Suicidal behavior can be classified into three categories: (1) suicidal ideation, which refers to thoughts of engaging in behavior that leads to the end of one’s life on purpose; (2) suicide planning, which is the formulation of a specific method that leads to the end of one’s life on purpose; and (3) suicidal attempts, which involve engaging in acts that potentially lead to self-injury and at least with some intent to die on purpose [3].

Dementia has been recognized as a global public health issue, as more than 55 million people live with dementia worldwide. Every year, approximately 10 million incident diagnoses are recorded, and current projections assume that by 2050, approximately 139 million people globally will live with dementia [4]. People with dementia not only are affected by cognitive decline but also experience various emotional problems [5–7]. Caregivers—predominantly family but also friends—provide a majority of dementia care, estimated to represent 40% of the total cost of dementia worldwide [8]. Several studies have shown that mental health aspects play a central role in the overall health of people with dementia [8]. For instance, caregivers of people with dementia have been reported to exhibit a greater risk of experiencing depression and anxiety than caregivers of patients with other medical conditions [9]. In essence, people with dementia and their caregivers are at risk of suicidal behaviors. In the context of caregivers of people with dementia, the prevalence of suicidal ideation range from 4.69% to 77.78%, while the prevalence of suicidal attempt range from 5.9% to 16.1% [10]. For suicidal behavior among people with dementia, the odds of suicidal ideation, suicidal attempt, and completed suicide have increased by 1.37-fold, 2.24-fold, and 1.28-fold, respectively compared to those of control subjects (age-matched or non-age-matched subjects without dementia) [11]. Despite the documented risk of suicidal behavior among people with dementia, the findings are still inconclusive, as some studies on suicidal behavior among people with dementia have not reported an increased risk of suicide [12]. Hence, exploring the possible reasons underlying the inconsistencies in suicidal risk among people with dementia is interesting.

According to a systematic review and meta-analysis of eight studies on suicidal behavior among caregivers of people with dementia, the risk factors contributing to increased suicidal behavior include depression, previous suicidal attempts, a sense of hopelessness, a history of comorbid psychiatric illness, lack of social support, demoralization, pain, and a feeling of being a burden on others [10]. Risk factors leading to suicidal behavior among people with dementia include being diagnosed with dementia at a younger age, having advanced dementia, feeling a loss of control, feeling a burden due to functional impairment, feeling lonely and being isolated [11].

Basically, the mental health of people with dementia and their caregivers is expected to exhibit a bidirectional effect in which the mental health of one group affects the mental health of another group and vice versa [13]. It would be interesting to explore whether the risk factors leading to suicidal behavior among caregivers of people with dementia as well as for people with dementia are both caregiver-related and care receiver-related, as management should then be tailored to include early identification and treatment of risk factors for both caregiver stress and neuropsychiatric symptoms in people with dementia.

In addition, although previous studies on the risk of suicidal behavior among people with dementia and their caregivers have identified numerous risk factors, none of these studies have analyzed and categorized the risk factors in a systematic way so that specific psychosocial interventions and special attention could be given to managing these risk factors appropriately to safeguard the mental well-being of both caregivers and people with dementia. To the best of our knowledge, although the mental health of people with dementia and their caregivers is expected to exhibit a bidirectional effect, none of the previous studies have identified risk factors that are associated with suicidal behavior in both caregivers and people with dementia. Moreover, to date, no narrative or systematic review has explored the risk factors for suicidal behavior in people with dementia and their caregivers within the same review or investigated the similarities and differences in the risk factors contributing to suicidal behavior among people with dementia and their caregivers.

Hence, the present systematic review addresses this research gap by: (1) exploring the prevalence and risk factors for suicidal behavior among these two target populations and (2) assessing the similarities and differences in the prevalence and risk factors for suicidal behavior between people with dementia and their caregivers.

## Methods

This systematic review was conducted according to the Preferred Reporting Items for Systematic reviews and Meta-Analyses (PRISMA) statement [14].

### Search strategies

An electronic search of published literature from 1950 to 2023 was carried out using major databases, such as Google Scholar, Web of Science, PubMed, Scopus, PsycINFO, EMBASE, the Cochrane Library, and Medline. Initially, a preliminary search was performed using keywords such as “dementia” and “suicide” OR “suicide and dementia” OR “suicide and people with dementia”, “caregivers of people with dementia” and “suicide” OR “suicide and caregivers of people with dementia”. In addition, hand searching was carried out in this review, resulting in a page-to-page review of the key journals that may have published studies on suicidal behaviors of people with dementia and their caregivers. The list of key journals in which the authors performed manual searching is listed in Additional file 1. Hand searching was also carried out for conference proceedings.

### Inclusion and exclusion criteria

The literature was eligible for review if it fulfilled the following inclusion criteria: (1) was published in English language peer review journals, including in-press articles; (2) was published in research articles, case reports, or case series; and (3) was related to the prevalence and associated factors of suicide among people with dementia and caregivers of people with dementia. The literature was excluded if (1) the preprint version of the research article was not peer reviewed and its content changed.

### Data extraction

Two independent reviews of the search results were carried out by two authors (MFILBA and NIS) during the title/abstract screening stage and full-text screening stage. Dual independent review of the search results was reported to increase the number of relevant studies identified for systematic review [15]. During screening for the inclusion of the articles for review, any discrepancies in the findings between the two authors were discussed and resolved. If there was difficulty resolving any discrepancies, the opinion of the 3rd author (MAM) was sought. The details of the search steps in this review in one of the major databases are described in Additional file 2.

Initially, during the data extraction, two authors (MFILBA and NIS) individually extracted information from the articles for cross-checking (the information extracted included (information about the article [author(s), year of publication, title, and DOI]; eligibility for review; methods [study type, participant recruitment and selection; study duration; and study quality]; participants [total number, sample size estimation and actual sample size; age, sex, ethnicity, country, diagnostic criteria, sociodemographics, types of dementia, and history of mental illness]; outcomes [outcomes and timepoint(s) collected and reported; instrument used for diagnosis and rating]; results [for each outcome of interest: look for sample size, missing data, estimation of effect with confidence intervals and *p* value; and subgroup analysis]; and others [funding source, key conclusion, references to other relevant studies, correspondence requirement, and miscellaneous comments by study authors]. After reviewing a few articles together, a consensus on what to extract from the articles was reached, and the work was split between the two authors. During individual data extraction, the authors remained in constant communication. Articles that were difficult to determine were discussed among the authors. The interrater reliability between the authors was computed as Cohen’s kappa (κ) using the following formula: κ = (p_0_ - pe)/(1- pe), where p_0_ = relative observed agreement among the raters and p_e_ = hypothetical probability of chance agreement. p_o_ = the degree to which both of the raters agreed with each other (both raters rated “Yes” or “No” divided by the total ratings), which was 0.8830; both raters agreed “Yes” = 151; both raters agreed “No” = 151; and the total number of ratings = 342. p_e_ = the sum of “Yes” divided by the total ratings, multiple with rater 2 rating as “Yes” divided by the total ratings and (rater 1 rating as “No” divided by the total ratings, multiple with rater 2 rating as “No” divided by the total ratings). Hence, Cohen’s kappa was good (κ = 0.77), with substantial agreement between the two raters. The authors then coded the study characteristics and findings into a database. The coded data included methodological characteristics (study design; participants’ sociodemographic characteristics, such as age and sex; sampling method; sample size estimation; study objectives; and outcome measures); study findings; and study limitations. The review was organized into the following categories: prevalence of suicide among people with dementia, prevalence of suicide among caregivers of people with dementia, associated factors of suicide among people with dementia, associated factors of suicide among caregivers of people with dementia, and differences in prevalence of suicide, associated factors and suicidal behavior between people with dementia and caregivers of people with dementia.

In addition, the risk of bias of the selected studies was assessed with the Risk of Bias Assessment Tool for Nonrandomized Studies (RoBANS). The RoBANS is a tool developed to assess the risk of bias in the findings of nonrandomized studies. The RoBANS consists of 6 domains: selection of participants, confounding variables, measurement of intervention (exposure), blinding of outcome assessment, incomplete outcome data and selective outcome reporting (table). The mean time needed to complete the RoBANS was shorter than that needed for another risk assessment tool, the Methodological Items for NOn-Randomized Studies (MINORS) (9.5 min [SD = 3.39] per study vs. 10.45 [SD = 3.54] per study). RoBANS exhibited good ratings in terms of user convenience for 3 items evaluated by reviewers (mean of 5.7 of 7 on the Likert scale; 0.81%). Additionally, the facial validity of 7 items evaluated by external experts was “fair” (mean 5.4 of 7 on the Likert scale), and all the experts recommended the use of this scale to assess the risk of bias in nonrandomized studies [16].

Initially, two authors (MFILBA and NIS) independently assessed the risk of bias in the selected studies. Then, any disagreements were discussed and resolved, and arbitration was performed by the third author (MAM).

## Results

### Characteristics of the selected studies

**Fig. 1:**
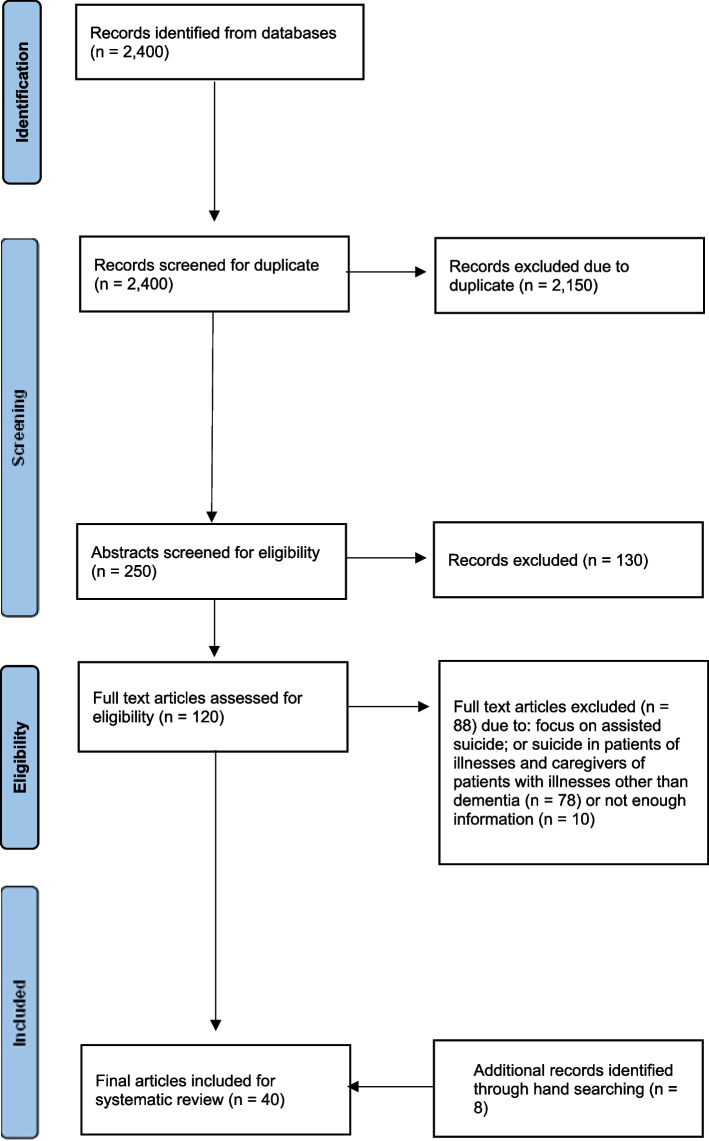
PRISMA flowchart summarizing the selection of research articles for this systematic review

An initial database search for titles and abstracts yielded 2,400 articles, but 2,150 articles were excluded as duplicates. Careful screening of the abstracts of the 250 remaining articles resulted in the exclusion of another 130 articles because they were not specific to suicide and/or were systematic reviews, narrative reviews, letters to the editor and editorials, commentaries, correspondences, or unpublished articles. Then, 120 full-text articles were screened for eligibility, and another 88 articles were excluded because they focused on assisted suicide or suicide in patients with illnesses and caregivers of patients with illnesses other than dementia or did not present enough information (studies that did not include study type, participant recruitment and selection, study duration, diagnostic criteria, sociodemographics, missing data, estimation of effects with confidence intervals and *p* values, or key conclusions). Hence, 40 articles were ultimately included in the review after another 8 additional articles were discovered through manual searching. The flow of the search findings is illustrated in Fig. 1.

The studies selected for review included 12 studies on suicidal behavior among caregivers of people with dementia and 28 studies on suicidal behavior among people with dementia. Six of the selected studies on suicidal behavior among caregivers of people with dementia were quantitative studies, 5 were qualitative studies, and 1 was an intervention study. All 26 selected studies on suicidal behavior among people with dementia were quantitative studies. The total combined sample size of studies on suicidal behavior among caregivers of people with dementia was 1,555,251 subjects (range from 9 subjects to 1,018,000 subjects), while the total combined sample size of studies on suicidal behavior among caregivers of people with dementia was 6,646,808 subjects (range from 24 to 2,667,987 subjects). None of the 40 selected studies mentioned the sampling method, except for the Valente et al. (2011), Lewis (2015) studies, and Choi et al. (2021) [17–19]. Similarly, all 40 studies did not calculate the estimated sample size needed to achieve the objectives of the studies. The characteristics, findings, and limitations of the selected studies are summarized in Table 1.

**Table 1:** Summary of the 40 selected articles for the systematic review

**Author(s)/ year/ country**	**Study design**	**Participants (N/ gender/ age)**	**Sampling method**	**SSE(Yes/No)**	**Study objective(s)**	**Outcomemeasures (Measures/ Intervention)**	**Findings**	**Limitations**
**Suicide among caregivers of people with dementia**								
**Hosaka and Sugiyama 2003** [20]	Intervention study	*N* = 20, all females from 47 to 66 years, 10 caretakers of vascular dementia and 8 caretakers of Alzheimer’s disease	NA	No	To investigate the effects of a 5-week structured group intervention on the immune function of caregivers of dementia patients.	POMS- to measure mood disteubance andGHQ-30- to assess other comorbidity	(1) Pre-intervention mean score for suicidal depression domain in GHQ-30 was 1.10 (SD = 1.48) and post-intervention at 0.85 (SD = 2.85). (2) Other findings: Depression mean score (POMS) was 16.1 (SD = 23.0) pre-intervention and 12.1 (SD = 12.6) at post-intervention. (3) 11 out of 20 subjects had no social support.	(1) Small sample size. (2) Subjects recruited from one centre.
**Shaji et al. 2003** [21]	Qualitative study	*N* = 17, 76% were females	NA	No	To assess the range of care arrangements, attitudes towards caregiving roles and sources of strain among caregivers of Alzheimer’s disease patients	Use of topic guide	(1) Depressed mood was reported by 16 (94%) of caregivers. Five caregivers had suicidal ideation and one had made an attempt. The caregiver who committed suicide had major depressive disorder. (2) Sources of caregiver strain: impairment in basic activities such as eating, dressing, bathing, and maintaining personal hygiene; incontinence; behavioral and psychological symptoms of dementia (BPSD). (3) Those caregivers who received help from others felt less stressed and appeared to be coping better. Better financial status, and other helpful adult women caregivers in the family were clearly helpful factors.	(1) Study did not consider saturation point for subject recruitment.
**Valente et al. 2011** [17]	Cross-sectional study	*N* = 137, 80.3% females	Convenient sampling	No	To investigate caregivers of dementia patients perceived health and to look into relationships with patients and caregivers’ sociodemographic and clinical data.	BAI, BDI, ZBI, and MBI	(1) 8.8% (*n* = 12) had death wishes. (2) Higher burden of care, severity of anxiety symptoms, severity of depression symptoms, emotional burnout, and depersonalization lead to higher odds of emotional problem.	(1) Cross-sectional design, no causal inference. (2) Non-probability sampling used. (3) Small sample size. (4) Sample recruited only from one center.
**O’Dwyer et al. 2012 **[22]	Online cross-sectional survey	*N* = 120, 89.2% female	NA	No	To gather preliminary evidence on suicidal ideation infamily carers of people with dementia.	RMBPC, FCSES, ADKS, SF-12 II, CESDS, BHS, GAI, LOT, ZBS, Brief COPE, DSSI, SBQ-R	(1) 26% of carers had contemplated suicide more than once in the previous year. Only half of these had ever told someone they might commit suicide and almost 30% said they were likely to attempt suicide in the future. (2) Carers who had contemplated suicide had poorer mental health, lower self-efficacy for community support service use and greater use of dysfunctional coping strategies. (3) When all factors were controlled, only higher severity of depression predicted presence of suicidal thoughts.	(1) Small sample size. (2) Cross-sectional study. (3) Sampling method used was convenient sampling. (4) Online survey limits generalizability of research findings.
**O’Dwyer et al. 2013 **[23]	Qualitative study	*N* = 9, 4 males and 5 females; 55.6% females	NA	No	To conduct an initial exploration of carers of dementia patients experiences of suicidality and identify factors associated with risk and resilience	A semi-structured interview guide. The questions focused on experiences and challenges of caring, participant approaches to managing stress and maintaining wellbeing, and experiences of suicidal ideation or suicide attempts.	(1) Three themes were identified in the data – ‘experiences of suicidal ideation’, ‘risk factors’ and ‘resilience’. Four of the nine participants had experienced suicidal thoughts and two had made preparations for a suicidal act. (2) Risk factors included pre-existing mental health problems, physical health conditions, and conflict with other family or care staff. (3) Factors positively associated with resilience included the use of positive coping strategies, faith, social support and personal characteristics.	(1) Study did not consider saturation point for subject recruitment. (2) Participants self-selected.
**Lewis, 2015 **[18]	Qualitative study	*N* = 101, 87% females (those who love one with dementia had passed away more than 10 years)	Purposive sampling	No	To discover a substantive theory that identifies the main problem that caregivers of loved ones with dementia face at the end of life and the basic social process by which they resolve that problem.	Grounded theory transcends description of data to conceptualize ideas that are substantive.	(1) Caregivers faced a concern of being trapped in an inescapable role. They felt bound to loved ones emotionally, mentally, and often physically. (2) Caregivers attempt to resolve this problem through a 5-stage basic social psychological process of rediscovering: (i) missing the past (many caregivers described their years of caregiving as “saying a long goodbye.”), (ii) sacrificing self (caregivers devote themselves to trying to minimize losses and control the “downward spiral” of their loved ones and ultimately sacrifice themselves), (iii) yearning for escape (ambiguity of prognosis toward the end-of-life period led to caregivers guessing when the end was near and seeing “no end in sight, death of care receiver seems to be the only way out), (iv) reclaiming identity (after caregivers reached the point of needing escape, they strategized ways to sustain themselves), and (v) finding joy (as a consequence of reclaiming themselves, caregivers were able to find true joy in their roles).	(1) Lack of diversity in demographic background. (2) Use of published memoirs as data may skewed the findings.
**Koyama et al. 2017 **[8]	Case-control study	*N* = 104, matched for age and gender, 58.7% females	NA	No	To compare the mental health of dementia caregivers with that of community residents and to clarify factors related to mental health problems in younger and older caregivers.	CESDS, SF-8, NPI, PSMS, LIADLS	(1) Both younger and older caregivers had significantly worse mental QOL than community residents, but were not more depressed. (2) Sleep problems were significantly more frequent in younger caregivers (39.1%) than in community residents. (3) Caregivers’ deteriorated mental QOL was associated with patients’ BPSD in younger caregivers and with dementia patients’ instrumental ADL and female gender in older caregivers.	(1) Small sample size. (2) Cross-sectional design. (3) Use different questionnaires to assess mental state of older and younger caregivers. (4) Sample recruited from a single center.
**Joling et al. 2018 **[24]	Longitudinal study	*N* = 192, 70.3% females, divided into three groups (suicidal thought, no suicidal thought, not assess)	NA	No	To explore thoughts of suicide, self-harm and death in dementia caregivers and investigates the characteristics that distinguish them from those without such thoughts.	MINI, CESDS, HADS-A, CRA, PMS, SSCQ	(1) Within 24 months, 76 caregivers reported symptoms of a potential depression and were further assessed for suicidal thoughts. (2) Nine carers (11.8%, 4.7% of the total sample) reported suicidal thoughts with three of those at multiple points. (3) Caregivers with suicidal thoughts had more severe depressive and anxious symptoms, had a lower sense of competence and mastery, felt less happy and experienced more health problems, less family support and more feelings of loneliness than caregivers who had not.	(1) thoughts of suicide, thoughts of self-harm and thoughts of death were grouped together. (2) Age and education differ between the groups of subjects.
**Anderson et al. 2019 **[25]	Qualitative study	*N* = 9 blogs	NA	No	To analyze a sample of blogs written by family caregivers of people with Alzheimer’s disease and related dementia to explore thoughts of suicide and homicide expressed by these caregivers.	Transcripts were analyzed in chronological order by two authors using codes created from the verbatim words used by the bloggers.	(1) Five themes related to thoughts of suicide and homicide by caregivers and people with ADRD were derived from the analysis: (i) end-of-life care (majority of caregivers wrote about what they described as “the long good bye”); (ii) thoughts of death and euthanasia by the person with ADRD (caregivers documented the person with ADRD’s thoughts and reflections on their own impending death); (iii) surrogate decision making (caregivers often wrote about concerns surrounding surrogate decision making); (iv) thoughts of suicide by the caregiver (caregivers expressed thoughts related to their own death); and (v) thoughts of homicide and euthanasia by the caregiver (caregivers in this sample of bloggers also wished for the care recipient’s death).	(1) May missed those who did not have access to internet. (2) May missed those who referred themselves as “carers” rather than “caregiver” in the blogs.
**Joling et al. 2019 **[26]	Longitudinal study	*N* = 6646 (first wave), 1582 (second wave and third wave); informal caregivers, 70.3% females	NA	No	To compare suicidal thoughts between non-caregivers and informal caregivers of people with a variety of conditions, in a large representative sample, and to identify significant risk factors.	CIDI version 3.0 (suicidal module)	(1) Thirty-six informal caregivers (2.9%) reported suicidal thoughts during the 4 year study period. (2) The difference between caregivers and non-caregivers (3.0%) was not significant. (3) Among caregivers, significant risk factors for suicidal thoughts included being unemployed, living without a partner, having lower levels of social support, having a chronic physical disorder, a mood disorder or an anxiety disorder, and having impaired social, physical and emotional functioning. These risk factors were also found in non-caregivers. (4) No caregiving-related characteristics were associated with suicidal thoughts.	(1) Small number of participants with suicidal thought, no multivariate analysis could be performed. (2) Participants asked to recall retrospective history of suicidal thought leading to recall bias.
**Kim et al. 2019 **[27]	Qualitative study	*N* = 18, Korean American caregivers, 83.3% females	NA	No	(1) To explore the caregiving experience of KA families of PWD and to understand how KA caregivers of PWD try to fulfill the gaps between their needs and available healthcare services for dementia care in the U.S. (2) To utilize this needs assessment for developing a community-based, caregiver-centered, and culturally appropriate dementia care education series for the KA community.	Semi-structured interview	(1) Four themes were identified: (i) challenges in finding resources (efforts to search for helpful resources, which were affected by multiple factors such as English proficiency, health insurance, financial status, knowledge of dementia, and attitude towards the illness), (ii) struggling with mental health issues (KA family caregivers’ struggle with several challenges related to dementia symptoms, their own emotions, health management, and family dynamics), (iii) traveling the path of acceptance (several caregivers discussed coming to terms with a new reality and putting efforts into providing the best possible care in the areas like nutrition and diet, exercise and activities, as well as communication strategies with compassion and love), and (iv) finding ways to survive (most caregivers reported self-care strategies such as walking, healthy eating, rest and sleep, medical check-ups, and hobbies).	(1) Recruitment in geographically limited location.
**Rosato et al. 2019 **[28]	Cross-sectional study	*N* = 1,018,000 people aged 25–74 years (130,816 caregivers; 110,467volunteers; and 42,099 engaged in both), not specific to dementia caregiver, 52.3% to 61.2% females	NA	No	(1) To compare the prevalence of poor mental health amongst volunteers and caregivers after adjustment for demographic and socio-economic factors; (2) To measure the risk of suicide amongst caregivers and volunteers, controlling for baseline health status and possible health selection effects; and (3) To determine if these prosocial activities reduce suicide risk for those with poor mental health.	Both definite suicides and deaths of undetermined intent were combined to define suicide. Sensitivity analyses were undertaken using just definite suicides.	(1) Intense caregiving was associated with worse mental health and volunteering with better mental health. (2) For those engaged in both activities, likelihood of poor mental health was determined by caregiving level. (3) There were 528 suicides during follow-up, with those engaged in both activities having the lowest risk of suicide. Engaging in either volunteering or caregiving was associated with lower suicide risk for those with good mental health at baseline but not for their peers with baseline poor mental health.	(1) Ethnicity background was limited to white.
**Suicide among people with dementia**								
**Lyness et al. 1992 **[29]	Cross-sectional study	*N* = 160, age 60 and above, 73.6% females	NA	No	To describe the psychopathological characteristics of elderly suicide attempters admitted to an inpatient psychiatric unit.	DSM-III	(1) Eighty percent of the attempters had a major depressive syndrome; (2) Dementia patients did not contribute to suicidal attempts.	(1) Cross sectional design. (2) Sample recruited from a single center.
**Florio et al. 1997 **[30]	Cross-sectional study	*N* = 683, 66.9% females, elderly patients referred to community-based aging and mental health service	NA	No	To determine whether elderly patients referred to community-based aging and mental health service who judged to be at risk for suicide differed from those persons judged not to be at suicide risk.	DSM-III, self-deisgned questionnaire	(1) Only 8% of subjects with suicidal risk had dementia. Dementia did not contribute to suicidal risk among the elderly.	(1) Self-designed questionnaire was used, except DSM III. (2) Subjects recruited from single location. (3) Cross-sectional design of the study.
**Rao et al. 1997 **[31]	Cross-sectional study	*N* = 118, all of whom were a cohort in a pre-existing epidemiological study of dementia who were community residents, 72% females	NA	No	To study the relationship between suicidal thinking and both cognitive impairment and depression.	CAMDEX, GDS, SSI	(1) Those with suicidal thinking showed higher CAMDEX depression scores, weaker strength of the wish to go on living, higher rates of expressing wish to die and higher rates of depressive illness and mixed DAT/multi-infarct dementia as primary psychiatric diagnoses. (2) No signi®cant associations between suicidal thinking and GDS scores, Alzheimer-type dementia alone, awareness of memory difficulties or severity of dementia.	(1) Small sample size. (2) Cross-sectional design.
**Rubio et al. 2001 **[32]	Retrospective case-control study	*N* = 28 elderly with completed suicide (case), 56 elderly who died naturally (control), age and gender matched; 39.3% females	NA	No	To determine if Alzheimer’s disease changes are overrepresented in elderly people committing suicide.	A modified Braak scoring system and semiquantitativeassessment of neurofibrillary tangles, amyloid deposition, Lewy bodies, and Lewy-associated neurites.	(1) The brains of individuals who committed suicide had higher modified Braak scores than those of matching control subjects. (2) The number of neurofibrillary tangles in CA1 was not an independent predictor of suicide status in the statistical analysis, although the distribution was more highly skewed among the cases. Hence, not indicative of dementia as cause of suicide.	(1) Small sample size. (2) Sample size of cases and controls not the same.
**Draper et al. 2003 **[33]	Cross-sectional study	*N* = 593 residents in 10 nursing home, 73.2% females	NA	No	To determine whether indirect self-destructive behaviors predict mortality in nursing home residents.	HBS, BEHAVE-AD, FASS, RCI, CIRS, EBASD, and the suicide item from the HRDS.	(1) Mortality was predicted by older age, male gender, lower level of functioning, lower levels of behavioral disturbance on the BEHAVE-AD, and higher scores on the HBS “passive selfharm” factor-based subscale, which includes refusal to eat, drink, or take medication. (2) Risk taking, active self-harm, and passive self-harm were postively correlated with behavioral pathology in Alzheimer’s disease.	(1) Lack of direct observational data (2) Cross-sectional design.
**Heun et al. 2003 **[34]	Case-control study	*N* = 67 Alzheimer’s disease patients, 109 elderly from general population, and 189 siblings; 62–82% females	NA	No	To compare the presence and symptomatology of depression between Alzheimer’s disease patient and age-matched non-demented subjects.	CIDI, MMSE	(1) Lifetime depressive symptoms were significantly more frequent in 76 AD patients than in 109 age-matched elderly from the general population. These 76 AD patients complained more about thinking and concentration disturbances, and less about depressed mood or appetite disturbance than the 298 non-demented participants matched for the lifetime presence of major depression (MD). (2) In agreement, the 29 patients comorbid for lifetime diagnoses of AD and MD reported less about depressed mood than the 114 age-matched elderly with MD only. (3) Feelings of worthlessness and suicidal ideas were related to the severity of cognitive decline.	(1) Since demented patients were recruited from a clinical population, there might be an overestimation of the prevalence of depressive symptoms compared to patients with AD in general population. (2) Analysis was based on retrospectively given information by demented and non-demented elderly.
**Peisah et al. 2007 **[35]	Case-control study	*N* = 143 community-dwelling suicide victims aged 65 years or more and 59 motor vehicle accident victims autopsies; 30.7% females	NA	No	To investigate prevalence of AD-related pathology in older suicide victims.	Senile plaques (diffuse and neuritic) a modified Bielschowsky (Garvey) silver technique, modified Braak score, plaque density was rated using the CERAD criteria, neuropathological rating was performed blind to the subjects’ clinical status.	(1) There were no significant differences in plaque score or neurofibrillary tangle staging between suicide and control groups. (2) None of the subjects with a history of dementia had neuropathologically confirmed AD.	(1) This retrospective study was limited, by the availability of tissue sections, to hippocampal and neocortical examination.
**Erlangsen et al. 2008 **[36]	Dynamic cohort study	*N* = 2,474,767 (all individuals age 50 + years living in Denmark); 52.8% females	NA	No	To examine the risk of suicide in persons diagnosed with dementia during a hospitalization and its relationship to mood disorders.	Outcome of interest is suicide. Relative risks are calculated based on person days spent in each stratum.	(1) 136 persons who previously had been diagnosed with dementia died by suicide. (2) Men and women aged 50–69 years with hospital presentations of dementia have a relative suicide risk of 8.5 and 10.8, respectively. Those who are aged 70 or older with dementia have a threefold higher risk than persons with no dementia. (3) The time shortly after diagnosis is associated with an elevated suicide risk. (4) The risk among persons with dementia remains significant when controlling for mood disorders. (5) 26% of the men and 14% of the women who died by suicide died within the first 3 months after being diagnosed, whereas 38% of the men and 41% of the women died more than 3 years after initial dementia diagnosis.	(1) Study only restricted to subjects diagnose with dementia during hospitalization.
**Purandare et al. 2009 **[37]	Retrospective case control study	*N* = 118 dementia patients died by suicide compared with *N* = 492 age and gender-matched non-dementia patients; 47% females	NA	No	To describe behavioural, clinical and care characteristics of people with dementia who died by suicide.	ICD-10	(1) The most common method of suicide in patients with dementia was self-poisoning, followed by drowning and hanging, the latter being less frequent than in controls. (2) Significantly fewer suicides occurred within 1 year of diagnosis in patients with dementia. (3) Patients with dementia were also less likely to have a history of self-harm, psychiatric symptoms and previous psychiatric admissions.	(1) This study is a survey of the clinical circumstances preceding suicide and unable to make causal inference. (2) The generalisability of findings is limited to patients with dementia in contact with mental health services, most likely patients with dementia with significant BPSD. (3) The clinicians who provided the information were not masked to patient outcome and this may have affected their response to certain questions.
**Qin, 2011 **[38]	Case-control study	*N* = 21,169 suicides in Denmark over a 17-year period with sex-age-time-matched population controls; 57% females	NA	No	To assess suicide incidence rate ratio (IRR) and population attributable risk (PAR) associated with various psychiatric disorders	ICD-10	(1) Suicide risk was significantly increased for persons with a hospitalized psychiatric disorder and the associated risk varies significantly by diagnosis and by sex and age of subjects. Recurrent depression and borderline personality disorder increase suicide risk the strongest while dementia increases the risk the least for both males and females.	(1) Cross sectional design.
**McCarthy et al. 2013 **[39]	Retrospective cross-sectional study	*N* = 281,066 from 137 nursing homes; 3.1% females	NA	No	To assess suicide rates up to 6 months following dischargefrom US Department of Veterans Affairs (VA) nursing homes.	ICD-10 Revised	(1) Suicide rates within 6 months of discharge were 88.0 per 100 000 person-years for men and 89.4 overall. (2) Dementia not associated with increase hazard ratio of suicide.	(1) Findings were derived from VA data and apply only to veterans who receive care from the VA health system. (2) Low proportion of women discharged from VA nursing home.
**Borges et al. 2015 **[40]	Cross-sectional study	*N* = 1992,	NA	No	To estimate if dementia and other mental disorders are associated with suicide ideation among the older people controlling for demographic and other suspected risk factors.	DSM-IV, ICD-10, CDRS, GMS–AGECAT Package	(1) Lifetime prevalence of suicide ideation of 13.5% and a 2-week prevalence of 4.2%. (2) Dementia plays a minor role on suicide ideation after the other variables were taken into account and its effect, if any, could be concentrated among those elders with lower severity scores of dementia.	(1) Cross-sectional design. (2) Limited set of variables to characterize suicide ideation and no information on the severity and persistency of suicide ideation. (3) Study cannot differentiate among types of dementia.
**Randall et al. 2014 **[41]	Population-based, propensity score–matched analysis	*N* = 2100 suicide deaths and 8641 attempted suicides. Three control subjects were identified for every case and matched on age, sex, income decile, region of residence, and marital status; 50.7% females	NA	No	To determine the degree of risk during the first year after diagnosis with a mental illness.	The Vital Statistics registry of the province was used to determine cases of suicide. Suicide attempts were determined through analysis of physician claims and hospital admission records. The presence of physician-diagnosed depression, anxiety disorders, substance use, schizophrenia, dementia, and other psychosocial disorders was determined using Manitoba RHA Indicators Atlas 2009.	(1) All disorders, except dementia, were independently related to death. All disorders were related to suicide attempts.	(1) Limited by the diagnostic coding used by physicians and hospitals, such as coding major depressive disorder and bipolar disorder under the same code. (2) Inability to adjust for factors that are not covered in the administrative data, such as stressful life events, childhood adversity, and similar nonmedical factors could not be measured and adjusted for in the analysis. (3) Inability to adjust for the severity of the disorder, the treatments used by the patients, and whether patients adhered to the treatments they were prescribed by their physicians.
**Nishida et al. 2015 **[42]	Retrospective case-control study	*N* = 24 posttroke depression deceased subjects, 11 of these had committed suicide, and the other 13 had not; 70.1% females	NA	No	To investigate the neuropathologic characteristics of poststroke depression (PSD) leading to suicide.	DSM-4, ICD-10 Revision, CDRS, immunohistochemistry using antibodies to phosphorylated tau, phosphorylated synuclein, A-amyloid, and B-crystallin	(1) Lesion type, size of stroke, and location of stroke were variable but did not differ significantly between the groups. (2) Alzheimer disease related pathology stages also did not differ between the groups.	(1) Small sample of patients with PSD, including those who had committed suicide. (2) Diagnosis and treatment of cases were provided by different neurologists and psychologists at different hospitals. (3) Unable to evaluate the subjects’ education level, which is a known influence risk for PSD, or other psychobiologic factors such as physical disability, ineffective coping skills, and lack of social resources.
**Matschke et al. 2018 **[43]	Retrospective case-control study	*N* = autopsies of 167 suicide dementia cases compared with age- and sex-matched controls who died of other cause. Each suicide was matched to one control according to sex and age within a range of 3 years; 33.3% females	NA	No	To investigate the prevalence of neurodegenerative changes in the brains of suicides of all ages in comparison with age- and sex-matched controls.	Semiquantitative analysis of neuritic plaques and neurofibrillary tangles visualized with silver stains; quantitative immunohistochemical analysis of β-amyloid load and counts of tau-positive neurofibrillary tangles and neuropil threads	(1) No effect of any parameter associated with the odds of committing suicide. On the contrary, after stratification for age, older suicide victims (over 48 years) showed lower β-amyloid loads when compared to controls in the univariate analysis. (2) In conclusion, neuropathological characteristics of Alzheimer’s disease and common tauopathies associated with age seem to be of limited relevance for suicides. (3) However, intact cognition when planning and carrying out complex acts may be of importance in the context of suicide.	(1) No access to any detailed clinical data, especially on the presence of depression, neuropsychiatric illness, or concerning antidepressant medications. (2) No opportunity to investigate the whole brain but only a defined subset of sections.
**Morgan et al. 2018 **[44]	Multiphase study	*N* = 4124 adults aged 65 years and older with a self-harm episode; 53% females	NA	No	To investigate the incidence of self-harm, subsequent clinical management, prevalence of mental and physical diagnoses, and unnatural-cause mortality risk, including suicide.	NICE Clinical Guideline CG16, ICD-10	(1) Overall incidence of self-harm in older adults aged 65 years and older was 4·1 per 10 000 person-years with stable gender-specific rates observed over the 13-year period. (2) Prevalence ratio of dementia in self-harm group to controls subsequently after index date not increased	(1) Some hospital-treated cases of selfharm will not have been reported to an individual’s GP and, therefore, will not have been captured. (2) Studies investigating suicide tend to underestimate because coroners might be reluctant to return a verdict of suicide more frequently in unnatural deaths of older people who might have strong religious affiliations, and levels of stigma surrounding suicide among this age group.
**Zucca et al. 2019 **[45]	Retrospective case-control study	*N* = 35 bvFTD patients and 25 controls; 56.7% females	NA	No	To determine the prevalence of suicidal ideation and attempts in bvFTD patients, evaluating possible risk factors for suicidality.	SSI, MMSE, CDR, FAB, AES-C, HDRS, HARS, PSS, BIS-11, BHS, neuroimaging investigations (brain MRI and 18-FDG PET)	(1) 40% of bvFTD patients had suicidal ideation in comparison to 8% of controls (*p* = .009). Four bvFTD patients have attempted suicide versus none control (*p* = .006). (2) BvFTD patients with suicide risk showed higher levels of anxiety, depression, stress and hopelessness than patients without suicide risk (*p* < .001). (3) Patients who attempted suicide were younger, and had a longer disease duration than those with only suicide ideation. (4) 40% of patients with parkinsonism presented high level of suicide ideation.	(1) Small sample size (2) Only study on bvFTD, not other types of FTD (3) bvFTD patients present a lower level of education in respect to controls.
**Ng et al. 2020 **[46]	Retrospective case-control study	*N* = 183 ADAD at-risk individuals (91 mutation carriers and 92 non-carriers); 61.2% females	NA	No	To study the frequency of suicidal ideation and its association with clinical and neurobiological correlates among cognitively intact ADAD at-risk individuals.	Suicide question from the UDS B6, GDS, informant-based NPI-Q, awareness of mutation status, neuropsychological assessments, and biological factors (genetic and cerebrospinal fluid)	(1) Twenty-six (14.20%) ADAD at-risk individuals (13 [14.28%] carriers and 13 [14.13%] non-carriers) had suicidal ideation. (2) The frequency of suicidal ideation did not differ between carriers and non-carriers. (3) Suicidal ideation was associated with higher GDS among all ADAD atrisk individuals. When stratified into mutation carrier status, non-carriers with suicidal ideation had higher GDS than carriers. (4) There was no statistically significant association between suicidal ideation and NPI-Q among ADAD at-risk individuals. (5) Awareness of mutation status, neuropsychological performances, and cerebrospinal fluid AD biomarkers were not associated with suicidal ideation among carriers and noncarriers.	(1) The single suicide question encompasses three components, which assess different levels of suicidal ideation. This may reduce the specificity in detecting the specific suicidal thought that may lead to an attempt. (2) GDS is originally designed to detect depressive symptoms among elderly individuals and may thus not be suitable for the younger participants in the DIAN cohort. (3) Cross-sectional design. (4) The inclusion of a comparator group, ideally of family members of individuals with sporadic AD, will be ideal to control for stress and caregiver burden, these data are unavailable for this study.
**Ortner et al. 2021 **[47]	Retrospective cross-sectional study	*N* = 157 dementia patients, 55.4% females	NA	No	To evaluate the prevalence of death wishes, suicidal ideation, and suicidal behavior of young and late onset dementia and to identify risk factors for suicidal ideation and behaviour.	CSSRS before diagnosis of dementia, immediately after diagnosis of dementia, and 30 days prior to interview	(1) 28% of the patients expressed suicidal ideation or behavior at some time after the onset of symptoms, and 9% of these within the month prior to the assessment. Two patients had attempted suicide after the onset of dementia. (2) There were no statistically significant differences between patients with and without suicidal ideations or behavior with regards to demographics or age at onset of dementia. (3) In patients with advanced dementia, Alzheimer’s disease (rather than frontotemporal lobar degeneration), better cognitive function, more severe psychological, behavioral, and physical symptoms, and a reduced quality of life were associated with the expression of suicidal ideation. (4) Patients with suicidal ideations in early stage of dementia stop to express them at advanced stages.	(1) Retrospective history from caregivers may lead to recall bias. (2) Small sample size. (3) Caregivers were asked to report on sucidal behavior of dementia patients, rather than the patients themselves which may lead to respondent bias.
**Alothman et al. 2022 **[48]	population-based case-control study	*N* = 594 674 patients with 580 159 (97.6%) were controls, 40 live control participants per suicide case were randomly matched on primary care practice and suicide date.	NA	No	To examine the association between a dementia diagnosis and suicide risk in the general population and to identify high-risk subgroups.	multiple linked electronic records from primary care, secondary care, and the Office for National Statistics in England from 2001 to 2019.	(1) Among those who died by suicide, 1.9% had a recorded dementia diagnosis. (2) There was no overall significant association between a dementia diagnosis and suicide risk. (3) However, suicide risk was significantly increased in patients diagnosed with dementia before age 65 years, in the first 3 months after diagnosis, and in patients with dementia and psychiatric comorbidity. (4) In patients younger than 65 years and within 3 months of diagnosis, suicide risk was 6.69 times higher than in patients without dementia.	(1) Diagnosis of dementia not confirmed clinically.
**Barak et al. 2002 **[49]	Retrospective case-control study	*N* = 1551 admission from 1991 to 2000 > 60 years old, divide into suicidal and non-suicidal patients (as controls)	NA	No	To examine the association between dementia and suicidal attempts.	DSM-4	(1) 22% diagnosed with dementia. (2) 7.4% of all AD patients were admitted immediately following a suicide attempt. (3) The index group (suicidal patients) differed from controls in Clinical Dementia Rating scores (*p* = 0.017) and higher frequency of previous suicide attempts (*p* = 0.022). (4) Lifetime psychopathology was not associated with higher rates of suicide attempts (*p* = 0.068). (5) Higher level of daily functioning and previous suicide attempts are associated with increased suicidal risk.	(1) Recruitment from only one center. Hence, findings not representative of dementia.
**Seyfried et al. 2011 **[50]	Case-control study	*N* = 294,952 dementia patients registered in Department of Veterans Affairs (VA) National Care Patient Database, 2.8% females	NA	No	To compare VA patients with dementia who committed suicide during the study period vs. those who did not by demographic characteristics, medical comorbidity, health care utilization and medication use variables. In addition, to examine the relationship between dementia severity and suicide and the methods used by those who killed themselves.	ICD-9 and ICD-10	(1) 8.17% of dementia patients died by suicide. (2) Increased suicide risk was associated with white race, depression, a history of inpatient psychiatric hospitalizations, and prescription fills of antidepressants or anxiolytics. (3) The majority of suicides occurred in those with new dementia diagnoses. (4) Firearms were the most common suicide method (73%).	(1) Study cohort predorminantly males. (2) Early dementia cases may not be diagnosed and identified.
**Tu et al. 2016 **[51]	Nationawide longitudinal cohort study	*N* = 1,189 patients aged ≥ 65 years who attempted suicide and 4,756 age- and sex-matched control subjects identified from the Taiwan National Health Insurance Research Database	NA	No	To investigate the risk of developing dementia in elderly people who had attempted suicide.	ICD-9	(1) Geriatric suicide attempt was associated with an increased risk of subsequent dementia. (2) Both patients aged between 65 and 79 years and patients aged ≥ 80 years who attempted suicide had an increased risk of developing dementia in later life, independent of depression and medical comorbidities.	(1) Only subjects who sought medical help and consultation were enrolled. Hence, subject may not represent the entire geriatric suicidal population. (2) The study did not include family history, personal lifestyle, environmental factors, and nutrition status.
**Annor et al. 2019 **[52]	Cross-sectional study	*N* = 141,592 persons with dementia in the 2013–2014 Medicare fee-for-service ADRD registry data, 67,706 persons with dementia who died during 2013–2016, 30.8% females	NA	No	To examine the characteristics, precipitants, and risk factors for suicide among persons with dementia.	Georgia Alzheimer’s Disease and Related Dementia (ADRD) registry	(1) Suicide rate among persons with dementia was 9.3/100,000 person-years overall and substantially higher among those diagnosed in the past 12 months. (2) Common precipitating factors were depressed mood (38.7%) and physical health problems (72.6%). (3) Being male, dementia diagnosis before age 65, and a recent diagnosis of dementia independently predicted suicide, but not depression or cardiovascular diseases.	(1) Recruitment only from a single state, hence findings may not represent the American dementia population. (2) Cross-sectional design. (3) Diagnosis of dementia not confirmed clinically. (4) Study did not control for all socio-demographic factors. (5) Those younger than 65 years may have missed as they had not gain Medicare service.
**Choi et al. 2021 **[19]	National Insurance Health survey for elderly	*N* = 528,655, those with dementia = 36,541, those without dementia = 36,541; 1:1 propensity-score matching using sex, age, comorbidities and index year, with follow-up throughout 2013	1:1 propensity-score matching	No	To investigate suicide risk in older adults within 1 year of receiving a diagnosis of dementia.	Mini-Mental State Examination score ≤ 26 and a Clinical Dementia Rating score ≥ 1 or a Global Deterioration Scale score ≥ 3 to identify those with dementia	(1) 46 suicide deaths (0.13%) during the first year after a dementia diagnosis. (2) Older adults with dementia had an increased risk of suicide death compared to those without dementia (AHR 2.57; 95% confidence interval [CI] 1.49–4.44). (3) Older adults with Alzheimer disease (AHR 2.50; 95% CI 1.41–4.44) or other/unspecified dementia (AHR 4.32; 95% CI 2.04–9.15) had an increased risk of suicide death compared to those without dementia. (4) Patients with dementia but without other mental disorders (AHR 1.96; 95% CI 1.02–3.77) and patients with dementia and other mental disorders (AHR 3.22; 95% CI 1.78–5.83) had an increased risk of suicide death compared to patients without dementia. (5) Patients with dementia and schizophrenia (AHR 8.73; 95% CI 2.57–29.71), mood disorders (AHR 2.84; 95% CI 1.23–6.53) or anxiety or somatoform disorders (AHR 3.53; 95% CI 1.73–7.21), respectively, had an increased risk of suicide death compared to patients with those conditions but without dementia.	(1) This study examined only elderly patients in South Korea, a population with a substantially higher suicide rate than the global population. Caution must be exercised when generalizing the results to populations with dissimilar backgrounds.
**Günak et al. 2021 **[53]	Nationwide longitudinal cohort study	*N* = 147 595: 21 085 patients with MCI, 63 255 with dementia, and 63 255 in the propensity-matched comparison group; 2.9% females	NA	No	To examine the association between diagnoses of MCI and dementia and suicide attempt and explore potential psychiatric moderators and to assess whether the association differs based on recency of diagnosis.	ICD-9, ICD-10	(1) 0.7% of patients with MCI and 0.6% of patients with dementia attempted suicide during follow-up, compared with 0.4% of patients without MCI or dementia. (2) After adjustment for demographic details and medical and psychiatric comorbidities, risk of suicide attempt was consistently highest for patients with a recent MCI or dementia diagnosis. (3) Risk associated with prior diagnosis was not significant. (4) No psychiatric comorbidity moderated the association between MCI or dementia and suicide attempt.	(1) Predorminant by male patients. (2) Study did not include potential risk factors such as social isolation, including loneliness, and assessment of brain injury. (3) Study did not focus on course or stage of illness itself.
**Holmstrand et al. 2021 **[54]	Cohort study	*N* = 1223 people with dementia from 8 European countries	NA	No	(1) To investigate the occurrence of suicidal ideation in older persons with dementia living at home, proxy-reported by their informal caregivers, in eight European countries. (2) To investigate factors associated with suicidal ideation, such as demographics, physical and mental health, type and stage of dementia, QoL, and psychotropic medication, and changes in suicidal ideation over time using 3-month follow-up data	Primary diagnosis of dementia and a Standardized Mini-Mental State Examination (SMMSE) score of < 24. Neuropsychiatric Inventory Questionnaire (NPI-Q); the SMMSE; the Cornell Scale for Depression in Dementia (CSDD); the Charlson Comorbidity Index (CCI); and the Quality of Life in Alzheimer’s Disease (QoL-AD) scale	(1) The occurrence of suicidal ideation in the participating countries varied between 6 and 24%. (2) Factors significantly (*p* < 0.0018) associated with suicidal ideation using bivariate analysis were: nationality, depressive symptoms, delusions, hallucinations, agitation, anxiety, apathy, disinhibition, irritability, night-time behaviour disturbances, anxiolytics and anti-dementia medication. (3) In multivariate regression analysis, country of origin, moderate stage of the dementia, depressive and delusional symptoms, and anti-dementia medication were significantly associated with suicidal ideation (*p* < 0.05). (4) Over time, suicidal ideation decreased from severe to mild or became absent in 54% of the persons with dementia.	(1) participants in the study were a very specific group of individuals on the margin of care, as they were deemed to require nursing home care within 6 months. (2) CSDD was not originally designed for use as a diagnostic instrument for depression in persons with dementia.
**Schmutte et al. 2022 **[55]	Nationwide retrospective longitudinal cohort	*N* = 2,667,987 older adults with newly diagnosed dementia, 62.7% females	NA	No	examined the risk of suicide in the first year following ADRD diagnosis relative to the general geriatric population.	ICD-10	(1) The suicide rate for the ADRD cohort was 26.42 per 100,000 person-years. (2) The overall standardized mortality ratio (SMR) for suicide was 1.53 with the highest risk among adults aged 65–74 years and the first 90 days following ADRD diagnosis. (3) Rural residence and recent mental health, substance use, or chronic pain conditions were associated with increased suicide risk.	(1) Diagnosis of dementia, not validated. (2) Study did not include potential risk factors such as lifetime history of self-harm, proximal stressful life events, social disconnection (e.g., marital status, loneliness), and access to lethal means. (3) Other causes of death may complicate detection of and contribute to underestimated counts of suicide deaths in older adults, particularly in the ≥ 75 years of age group.

*SSE* Sample size estimation, *POMS* Profile of Mood State, *GHQ-30* General Health Questionnaire-30, *BDI* Beck Depression Inventory, *BAI* Beck Anxiety Inventory, *ZBI* Zarit Burden Interview, *MBI* Maslach Burnout Inventory, *KA* Korean American, *PWD* People with dementia, *DSM III* Diagnostic and Statistical Manual for Mental Disorders 3^rd^ edition, *ICD 10* International Classification of Diseases 10^th^ Edition, *bvFTD* Behavioral variant of frontotemporal dementia, *RMBPC* Revised Memory and Behavior Problems Checklist, *FCSES* Fortinsky Caregiver Self-efficacy Scale, *ADKS* Alzheimer’s Disease Knowledge Scale, *SF-12 II* 12-item Short Form Health Survey Version 2, *CESDS* Center for Epidemiologic Studies Depression Scale, *BHS* Beck Hopelessness Scale, *GAI* Geriatric Anxiety Inventory, *LOT* Life Orientation Test, *ZBS* Zarit Burden Scale, *DSSI* Duke Social Support Index, *SBQ-R* Suicidal Behaviors Questionnaire-Revised, *SF-8* Health related quality of life short-form health survey, *NPI* Neuropsychiatric Inventory, *PSMS* Physical Self-Maintenance Scale, *LIADLS* Lawton Instrumental ADL Scale, *MINI* Mini International Neuropsychiatric Interview, *HADS-A* Hospital Anxiety and Depression Scale—Anxiety, *CRA* Caregiver Reaction Assessment, *PMS* Pearlin Mastery Scale, *SSCQ* Short Sense of Competence Questionnaire, *ADL* Activity of daily living, *QOL* Quality of life, *BPSD* Behavioral and psychological symptoms of dementia, *CIDI* Composite International Diagnostic Interview, *DSM* Diagnostic and Statistical Manual for Mental Disorders, *ICD* International Classification of Diseases, *CAMDEX* Cambridge Examination for Mental Disorders of the Elderly, *GDS* Geriatric Depression Scale, *SSI* Scale for Suicidal Ideation, *HBS* Harmful Behaviors Scale, *BEHAVE-AD* Behavioral Pathology in Alzheimer’s Disease Rating Scale, *FASS* Functional Assessment Staging Scale, *RCI* Resident Classification index, *CIRS* Cumulative Illness Rating Scale, *EBASD* Even Briefer Assessment Scales for Depression, *HDRS* Hamilton Depression Rating Scale, *MMSE* Mini Mental State Examination, *CDRS* Clinical Dementia Rating Scale, *GMS-AGECAT* Geriatric Mental State–Automated Geriatric Examination for Computer Assisted Taxonomy, *DRS* Depression Rating Scale, *UDS* Uniform Data Set B6, *NPI-Q* Informant-based neuropsychiatric inventory questionnaire, *CDRS* Clinical Dementia Rating Scale, *FAB* Frontal Assessment Battery, *AES-C* Apathy Evaluation Scale-Clinician Version, *HDRS* Hamilton Depression Rating Scale, *HARS* Hamilton Anxiety Rating Scale, *PSS* Perceived Stress Scale, *BIS-11* Barratt Impulsiveness Scale, *BHS* Beck’s Hopelessness Scale, *ADAD* Autosomal dominant Alzheimer’s disease, *AD* Alzheimer’s disease, *CSSRS* Columbia-Suicide Severity Rating Scale, *MCI* Mild cognitive impairment, *ARDS* Alzheimer’s disease or related dementias, *AHR* Adjusted hazard ratio

### Bias assessment of the selected studies

**Fig. 2:**
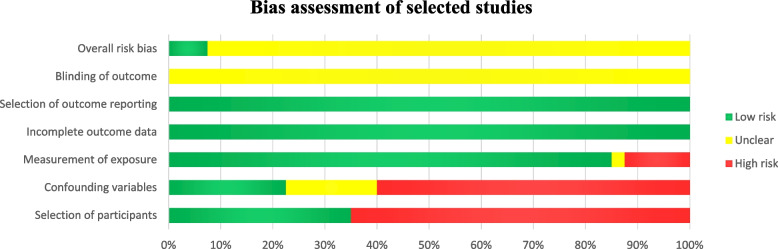
Risk of bias assessment of the selected studies using the Risk of Bias Assessment Tool for Non-randomized Studies (RoBANS)

For the assessment of the risk of bias of the selected studies using the RoBANS, the findings are summarized in Fig. 2 and Additional file 3. Regarding bias arising from the selection of participants (65%) and confounding factors (60%), more than half of the selected studies had a high risk of bias, whereas most of the selected studies with a low risk of bias investigated suicidal behavior among people with dementia. In contrast, with regard to bias arising from inadequate measures of exposure, the majority of the selected studies had a low risk (85%). In terms of inadequate blinding of outcome assessment, the risk of bias of all the selected studies was unclear because the studies did not assess this outcome. Due to inadequate handling of incomplete outcome data and bias caused by selective outcome reporting, all the selected studies had a low risk of bias, as there was no missing data. According to the overall assessment of risk of bias, 92.5% of the selected studies had an overall risk of bias, as they had a low risk of bias for at least two items in the RoBANS, but the risk of bias for other items was high or unclear (Fig. 2).

### Suicidal behavior among caregivers of people with dementia

Only two out of the twelve selected studies had relatively larger sample sizes (with a sample size of more than 1000 subjects) [26, 28]. Almost all the selected studies included a larger proportion of female subjects, ranging from 55.6% to 100%. The prevalence of suicidal behavior among caregivers of people with dementia in selected cross-sectional quantitative studies ranged from 4.7% to 26% [17, 22, 28]. However, in large quantitative studies involving sampling at the national level, the prevalence of suicidal behavior reported among caregivers of people with dementia ranged from 0.19% to 2.9% [26, 28]. The suicidal behaviors exhibited included suicidal thoughts, death wishes, and contemplating suicide [17, 18, 21, 22, 24, 25, 28]. Despite having suicidal thoughts, only half of the caregivers reported that they may commit suicide, and 30% of the caregivers disclosed that they may inform someone if they plan to attempt suicide in the future [22].

Regarding the thoughts and emotional responses of the caregivers toward caring for people with dementia, the selected qualitative studies identified several themes, which included concern about being trapped in an unescapable role as a caregiver, providing end-of-life care to one’s loved ones, thinking about impending death and wish for euthanasia, the caregiver’s role in surrogate decision making on behalf of one’s care receiver, experience of suicidal thoughts of the caregiver, thoughts of homicide and euthanasia of the caregiver toward the care receiver, challenges finding useful resources by the caregiver to facilitate caregiving, struggle of the caregiver with mental health issues (such as emotional disturbances, health management and family dynamics), acceptance of the caregiver’s role and attempt to do their best caring for the family member with dementia, and discovery of a strategy to survive the caregiver’s role (such as engaging in health diet, rest and sleep, walking, hobbies, and continuing medical checkup) [18, 23, 25, 27].

The most significant risk factor led to the occurrence of suicidal behavior among caregivers of people with dementia was the presence of depression [17, 20–22, 24, 26]. The prevalence of depression reported among caregivers of people with dementia with suicidal behavior ranged from 39.6% to 94% [21, 24]. In addition, other significant risk factors that predispose individuals to suicidal behavior among caregivers of people with dementia included increased severity of anxiety symptoms [17, 24, 26], preexisting physical health conditions [24–26], preexisting mental health problems [23, 28], and absence of social and family support [24, 26]. The frequencies of the risk factors that contribute to the occurrence of suicidal behavior among caregivers of people with dementia identified in the selected studies are summarized in Table 2.

**Table 2:** The frequency of risk factors which contribute to occurrence of suicidal behavior among people with dementia and their caregivers identified in the selected studies

	**Frequency of being investigated**
**Risk factors associated with suicidal behavior in both people with dementia and their caregivers**	
(1) Higher severity of depression and anxiety	• In people with dementia: 7 studies ([31, 34, 45, 46, 50, 52, 54]) • In caregivers of people with dementia: 6 studies ([17, 20–22, 24, 26])
(2) Pre-existing mental health problem	• In people with dementia: 2 studies [19, 48] • In caregivers of people with dementia: 2 studies [23, 28]
(3) Pre-existing physical health condition	• In people with dementia: 1 study [52] • In caregivers of people with dementia: 3 studies ([23, 24, 26])
(4) Presence of or more severe behavioral and psychological symptoms of dementia (BPSD)	• In people with dementia: 1 study [47] • In caregivers of people with dementia: 1 study [21]
**Risk factors associated with suicidal behavior only in people with dementia**	
(1) Shortly or within 3 months after diagnosis of dementia	7 studies [36, 47, 48, 50, 52, 53, 55]
(2) Younger age of diagnosis (50 to 69 years old)	4 studies [36, 45, 48, 52]
(3) History of inpatient psychiatric hospitalization	3 studies [37, 38, 50]
(4) Alzheimer’s disease alone	2 studies [19, 47]
(4) Passive self-harm	2 studies [33, 37]
(4) Better cognitive function	2 studies [43, 47]
(4) Advanced dementia	2 studies [47, 49]
(8) Higher thought and concentration disturbance	1 study [34]
(8) Worsening of cognitive decline	1 study [34]
(8) Mixed Alzheimer’s disease and vascular dementia	1 study [31]
(8) Lower degree of wish to live	1 study [31]
(8) Greater degree of wish to die	1 study [31]
(8) Reduced quality of life	1 study [47]
(8) Stress and hopelessness	1 study [45]
(8) Delusional symptoms	1 study [54]
(8) Antidementia medication prescription	1 study [54]
(8) Higher level of daily functioning	1 study [49]
(8) Previous suicidal attempts	1 study [49]
(8) Recent mild cognitive impairment	1 study [53]
(8) Living in rural area	1 study [55]
(8) Chronic pain	1 study [55]
(8) Substance use	1 study [55]
(8) Comorbid Parkinson’s disease	1 study [45]
(8) Prescription of antidepressant and anxiolytic medication	1 study [50]
(8) Moderate dementia	1 study [54]
**Risk factors associated with suicidal behavior only in caregivers of people with dementia**	
(1) Absence of social and family support	2 studies ([24, 26])
(2) Emotional burnout	1 study [17]
(2) Depersonalization	1 study [17]
(2) Lower self-esteem	1 study [22]
(2) Dysfunctional coping	1 study [22]
(2) Higher burden of care	1 study [17]
(2) Insomnia	1 study [8]
(2) Feeling lonely	1 study [24]
(2) Lower sense of competence and mastery	1 study [24]
(2) Poor mental health	1 study [22]
(2) Unemployment	1 study [26]
(2) Living without a partner	1 study [26]
(2) Impaired social, physical and emotional function	1 study [26]
(2) Care receiver related factors (impairment in basic activities such as eating, dressing, bathing, and maintaining personal hygiene; incontinence)	1 study [21]

### Suicidal behavior among people with dementia

Most of the selected studies consisted of a larger proportion of female subjects, except for nine studies, which consisted of more male subjects [19, 32, 35, 37, 39, 43, 50, 52, 53]. In most of the studies, the proportion of female subjects ranged from 52.8% to 82.0% [29–31, 33, 34, 36, 38, 40–42, 44–47]. Among the selected studies, only 13 studies specified the types of dementia diagnosed among the subjects; 11 studies examined subjects with Alzheimer’s disease [19, 29, 32–35, 42, 43, 46, 49, 55], while only one study investigated mixed Alzheimer’s disease and vascular dementia and frontotemporal dementia [31, 45].

Among the included studies that reported the prevalence of suicidal behavior among people with dementia, the prevalence ranged from 0.005% to 40% [19, 29, 42, 44–49, 51, 54]. The suicide rates reported in two included studies among people with dementia [52, 55] were 9.3 per 100,000 persons per year and 26.4 per 100,000 persons per year. However, the risk of suicidal behavior among people with dementia was inconclusive, as 17 selected studies reported the risk of suicidal behavior among people with dementia [19, 33, 34, 36, 37, 45–47, 49–55], but 11 selected studies did not [29, 30, 32, 35, 39–44, 48]. Among studies with relatively large sample sizes (more than 1000 subjects), only nine reported an increased risk of suicidal behavior [19, 36, 49–55], whereas four studies found no association between the risk of suicidal behavior and dementia [37, 38, 40, 48].

The suicidal behaviors reported included deliberate self-harm, suicidal ideation, and suicidal attempts (such as self poisoning, drowning, hanging, and the use of firearms) [31, 33, 34, 36, 37, 45–47, 49, 50, 52]. Six large-scale population-based studies indicated that the risk of suicidal behavior was greater within the first 3 months to 1 year after diagnosis and in people diagnosed with dementia before the age of 65 years [36, 48–50, 52, 53].

Two of the most significant risk factors for suicidal behavior among people with dementia were greater severity of depression and anxiety [31, 34, 45, 46, 50, 52, 54] and shortly or within 3 months of being diagnosed with dementia [36, 47, 48, 50, 52–54]. In addition, the other significant risk factors contributing to suicidal behavior among people with dementia included younger age at diagnosis (within 50 to 69 years old) [36, 45, 48, 52], history of inpatient psychiatric hospitalization [37, 38, 50], diagnosis of Alzheimer’s disease alone [19, 47], passive self-harm such as refusal to eat, drink, or take medication [33, 37], presence of comorbid psychiatric illnesses (such as mood disorders, schizophrenia, somatoform disorders and anxiety disorders) [19, 48], better cognitive function [43, 47], and advanced dementia [47, 49]. The frequencies of the risk factors that contributed to the occurrence of suicidal behavior among people with dementia, as identified in the selected studies, are presented in Table 2.

## Discussion

This comprehensive systematic review summarized the prevalence and risk factors associated with the occurrence of suicidal behavior and the differences between people with dementia and their caregivers.

### Prevalence of suicidal behavior in people with dementia and their caregivers

There were a few differences in the prevalence and risk of suicidal behavior between people with dementia and their caregivers. First, there was significant and clear evidence from selected studies that the risk of suicide was associated with caregivers of people with dementia (all selected studies indicate suicidal risk among caregivers of people with dementia). In contrast, evidence of the risk of suicide among people with dementia has been inconclusive (only 17 out of 28 studies indicated the risk of suicide among people with dementia). Notably, there were wide ranges of prevalence rates for both caregivers (4.7% to 26%) and people with dementia (0.005% to 40.0%), which is indicative of heterogeneity among the selected studies, which differ in methodology and causes of dementia. This may be a possible reason for the discrepancy between the association of suicidal behavior in people with dementia and that in their caregivers. Another possible reason for the inconclusive risk of suicidal behavior among people with dementia is that suicidal risk involves complex acts and include planning for suicide. Commonly, people with Alzheimer’s disease at an earlier stage after diagnosis and at a younger age (less than 65 years old) may have intact cognition, characterize by lesser cognitive decline and more intact daily functioning, could have higher ability to carry out complex act such as attempted suicide and planning for suicide, leading to higher suicidal risk among this group of people with dementia [29, 33, 40, 43, 48–50, 52, 53]. Moreover, the risk factors for suicidal behavior among people with dementia (lower degree of wish to live, greater degree of expression of wishing to die, greater severity of depression and anxiety symptoms, and stress and hopelessness) also pinpoint toward a greater risk of suicidal behavior among those with more intact cognitive function [31, 40, 45, 50].

Second, with reference to selected studies with larger population-based sample sizes (*n* > 100,000), the prevalence of suicidal behavior is relatively greater among people with dementia (0.005% to 8.17%) [19, 36, 49, 50, 52, 53] than among caregivers of people with dementia (0.19% to 2.9%) [17, 26]. However, larger-scale population-based studies that report data on the prevalence of suicidal behavior are still scarce; there are only four studies among people with dementia and two studies among caregivers. Hence, additional large-scale studies are needed to determine the prevalence of suicidal behavior more reliably among people with dementia and caregivers before a definitive conclusion can be drawn.

### Risk factors for suicidal behavior in caregivers of people with dementia

The risk factors for suicidal behavior among caregivers of people with dementia could also be classified into four main themes: high caregiver burden (unemployment, intense caregiving, lower sense of competence and mastery, impaired basic activities of daily living and the presence of BPSD among care receivers) [17, 21, 24, 26]; greater emotional burnout (greater severity of depression and anxiety symptoms, depersonalization leading to greater odds of emotional problems and the use of dysfunctional coping) [17, 20–22, 24, 26]; lack of social support (absence of social and family support, lower self-efficacy in seeking assistance from community service, conflict with family or care staff; feeling of loneliness, living without a partner, and issuing with family dynamics) [24, 26]; and history of preexisting illness (preexisting mental illness, mood disorders, and chronic physical illness) [17, 21, 25].

Among these risk factors categories, greater emotional burnout and history of preexisting illness stood out as the commonly identified risk factors of suicidal behavior among caregivers of people with dementia in the selected studies. These two categories of risk factors have also been reported as common risk factors among caregivers of people with dementia in other systematic and scoping reviews [10, 56]. The risk factors of suicidal behavior among caregivers of people with dementia will be discussed in more details later.

### Risk factors for suicidal behavior in people with dementia

Similarly, risk factors for suicidal behavior among people with dementia may also be categorized into emotion-related factors (higher severity of depression and anxiety, passive self-harm, presence of comorbid mood disorder, schizophrenia, somatoform disorder or anxiety disorder, history of inpatient psychiatric hospitalization, lower degree of wish to live, greater degree of wish to die, stress and hopelessness, delusional symptoms, previous suicidal attempts, more severe behavioral and psychological syndrome of dementia, and prescription of antidepressant and anxiolytic medication) [19, 31, 34, 37, 38, 45–50, 52, 54], dementia-related factors (shortly or within 3 months after diagnosis of dementia, younger age of diagnosis, Alzheimer’s disease alone, better cognitive function, advanced dementia, higher thought and concentration disturbance, worsening of cognitive decline, reduced QoL, mixed Alzheimer’s disease and vascular dementia, antidementia medication prescription, higher daily functioning, recent mild cognitive impairment, comorbid Parkinson’s disease, and moderate dementia) [19, 23, 31, 34, 36, 43, 45, 47–50, 52, 55], physical health-related factors (history of physical health problem and chronic pain) [52, 55], and psychosocial factors (living in rural areas and substance use) [55].

Among these risk factors categories, emotion and dementia-related factors were identified as the commonly identified risk factors of suicidal behavior among people with dementia in the selected studies. These two categories of risk factors have also been reported as common risk factors among people with dementia in other literature and systematic reviews [11, 57]. The risk factors of suicidal behavior among people with dementia will be discussed in more details later.

### Communalities and differences in the risk factors for people with dementia and their caregivers

A few risk factors were identified as risk factors contributing to suicidal behavior in people with dementia and their caregivers, such as greater emotional burnout (presence of and greater severity of depression and anxiety), which was the most commonly reported risk factor; preexisting mental health problems; preexisting physical health conditions; and the presence of or more severe behavioral and psychological symptoms of dementia (BPSD) (Table 2).

In the context of caregivers of people with dementia, the burden of caregiving creates mixed feelings among caregivers, contributing to a dilemma between carrying out the responsibility to provide end-of-life care to the care receiver with dementia and the homicidal thought of death of the care receiver. Hence, to end years of suffering because being bound to the role of a caregiver may subsequently contribute to depression and anxiety and the risk of carrying out suicidal behavior among caregivers of people with dementia [18, 25].

Major depressive disorder and anxiety among elderly people presented with pertinent deficits in processing speed or working memory and executive dysfunction, which may increase the risk of developing Alzheimer’s disease. Moreover, late-stage depression may be linked to hippocampal atrophy and generalized ischemia affecting the frontostriatal region of the brain, contributing to cognitive impairment, which may hasten the development of Alzheimer’s disease. Cognitive impairment in late-life depression could increase the risk of developing suicidal behavior [58]. Hence, these factors may explain how greater severity of depression and anxiety may contribute to higher risk of suicidal behavior among people with dementia.

Essentially, people with dementia and depression who present with passive self-harm would require urgent treatment and care, as they may harbor a high risk of presenting with suicidal behavior. Moreover, self-harm is well recognized as an early warning sign of suicide [59].

Preexisting physical health conditions and preexisting mental health problems were also common risk factors leading to suicidal behavior among caregivers of people with dementia. As anticipated, our findings consistently indicated that the caregiving role could predispose individuals to exacerbation of preexisting physical health and mental health conditions, consequently leading to an increased risk of developing suicidal behavior [23]. Similarly, in people with dementia, the presence of preexisting mental health problems (such as schizophrenia, mood disorders, somatoform disorders, or anxiety disorders) and comorbid physical illnesses (such as diabetes mellitus, cancer, ischemic heart disease, stroke, chronic kidney disease, or Parkinson’s disease) could worsen the risk of suicidal behavior [19, 48]. This should prompt clinicians to carefully screen people with dementia with comorbidities for risk of suicide. However, it remains unclear whether preexisting mental and physical illnesses play a predominant role as risk factors for dementia and suicidal behavior or as mediators in the relationship between dementia and suicidal behavior, or both, which may warrant further investigation.

In addition, greater severity of BPSD also increased the risk of suicidal behavior in people with dementia and their caregivers. Among the common BPSD experienced by people with dementia were agitation and sleep disturbance. Agitation arises when a person with dementia (a care receiver) experiences some unmet needs (such as a lack of attention received from the caregiver and inadequate interaction between the caregiver and the person with dementia), which creates a significant degree of caregiver burden and may ultimately contribute to the risk of suicidal behavior among caregivers. Similarly, sleep disturbance in people with dementia may contribute to or exacerbate sleep deprivation in caregivers, leading to emotional burnout and in turn increasing the risk of suicidal behavior among caregivers [60]. In the context of people with dementia, more severe BPSD may enhance perceived burdensomeness and diminish quality of life, leading them to value their life as less worth living than people with better quality of life [47].

Despite some commonalities in the risk factors contributing to suicidal behavior between people with dementia and their caregivers, there were some notable differences in risk factors between the two groups. Risk factors associated with suicidal behavior among caregivers of people with dementia were both self-related and related to caring for those people with dementia, whereas risk factors in people with dementia were self-related factors and not related to care received from caregivers.

There were several common risk factors that were associated with suicidal behavior only among people with dementia. Intriguingly, shortly after or within 3 months of diagnosis with dementia and young age at diagnosis of dementia (aged 50 to 69 years) were among the most common risk factors associated with suicidal behavior among people with dementia. Basically, individuals who have recently been diagnosed with dementia are still in a state where they have the cognitive capability and functioning to perceive their deficits and are still capable of planning and executing suicidal behaviors [31, 40, 45, 50]. Hence, people recently diagnosed with dementia are at higher risk of exhibiting suicidal behavior. Similarly, people who are diagnosed with dementia at a relatively younger age (50 to 69 years) carry a higher risk of presenting with suicidal behavior, as they may anticipate and must endure worsening and persistent loss of functioning for a long time in relation to their life expectancy, which will hamper their productive years and their responsibility to take care of their family. This experience of perceived burdensomeness leads to a decreased ability to accept and adjust to having to live with dementia, ultimately resulting in suicidal behavior [47]. Our findings also revealed that a history of psychiatric inpatient admission; the presence of Alzheimer’s disease alone; and the presence of passive self-harm, such as refusal to eat, drink and take medication, worsened the mental health of people with dementia and contributed to the development of suicidal behavior. Hence, people with dementia and the above risk factors need to be screened for suicide risk, and special attention is needed from treating clinicians.

In addition, our findings also pinpoint the importance of facilitating caregivers to maintain a sufficient degree of social and family support. Conventionally, social and family support are vital protective factors against depression [61]. The absence of social and family support may worsen depression and increase the risk of developing suicidal behavior among caregivers of people with dementia.

### Other notable findings

Most of the selected studies on the suicidal behavior of people with dementia and their caregivers involve a larger proportion of female participants. In the context of gender preponderance, the proportion of female caregivers of people with dementia is greater than that of male caregivers (ratio of 2:1), who are usually spouses or daughters of dementia patients [62]. This explained the larger proportion of female subjects in almost all of our selected studies. Similarly, the larger proportion of female dementia subjects reported in our review also confirmed the finding that females are more prone to dementia, particularly Alzheimer’s disease [63].

### Limitations

There were a few limitations to be considered in the selected studies on the suicidal behavior of caregivers of people with dementia. First, most of the selected studies had small sample sizes, except for two studies [17, 26]. Second, most of the selected studies were cross-sectional studies, except for two studies [24, 26]. Hence, causal inference of the risk factors identified for the occurrence of suicidal behavior among caregivers of people with dementia could not be deduced. Third, although there were two selected studies with large sample sizes, these studies were limited by retrospective recall of suicidal behavior, leading to the risk of recall bias, a small number of subjects with suicidal behavior [26], and a lack of diverse demographic backgrounds [18].

Due to the limitations of the selected studies on the suicidal behavior of people with dementia, most of the studies were cross-sectional or case‒control studies, and causal inference of the risk factors for the occurrence of suicidal behavior could not be made. In addition, six selected studies examined the autopsy findings of people with dementia who died due to suicide; these studies involved retrospective history of suicidal behavior and were prone to recall bias. Moreover, more than half of the selected studies recruited subjects from a single center; hence, the findings cannot be generalized to the entire dementia population. Finally, more than half of the studies did not specifically recruit subjects with dementia.

This systematic review has several limitations that must be considered. First, this narrative review did not include research articles, case reports, or case series published in languages other than English. Hence, we may have missed research findings that were reported in other languages. Second, this systematic review did not include preprint versions of the research articles; hence, we may have missed the latest research findings, which were still in the preprint version.

### Clinical implications

The findings of this systematic review should alert clinicians to pay special attention to screening caregivers of people with dementia who may be at high risk of developing suicidal behavior, such as those with greater severity of depression and anxiety, preexisting physical health and mental health conditions, and poor social and family support. Similarly, treating clinicians should be prepared to screen people with dementia for suicide risk, particularly those with more severe depression and anxiety, especially those with passive self-harm; those diagnosed with dementia within 3 months; especially those with better cognitive function; younger patients diagnosed with dementia (with age 50 to 69 years); and those with comorbid mental illness and a history of inpatient psychiatric hospitalization. Hence, people with dementia and their caregivers with a high risk of suicide should receive cognitive-focused control intervention and interpersonal psychotherapy to alleviate their risk of suicide [63, 64].

However, it remains inconclusive whether the specific cause of dementia could increase the risk of suicidal behavior among people with dementia, as the presence of Alzheimer’s disease alone and having a mixed diagnosis of both Alzheimer’s disease and vascular dementia have been reported to increase the risk of suicidal behavior. In addition, the selected studies in this review did not mention which types of suicidal behavior are more common among caregivers and people with dementia. These research gaps should be explored further in future studies.

## Conclusions

This systematic review pinpointed the definitive risk of suicidal behavior as a consequence of the caregiving role of caregivers of people with dementia, while the risk of suicidal behavior among people with dementia still remains inconclusive. Large population-based studies have revealed that the prevalence of suicidal behavior among people with dementia is greater than that among caregivers. There were a few risk factors that were associated with suicidal behavior in people with dementia and their caregivers, such as the presence and greater severity of depression and anxiety, preexisting mental illness and physical illness, and greater severity of BPSD among people with dementia. Hence, treating clinicians should carefully manage these risk factors to safeguard the mental health of people with dementia and their caregivers. We also recommend that clinicians conduct early suicide risk screening among people with dementia during the early phase of dementia, as intact cognitive function, complex acts and planning may increase the risk of suicidal behavior. Despite the potential implications of our review findings, the overall risk of bias of the majority of the selected studies was unclear, as a larger proportion of the selected studies had a high risk of bias due to inadequate selection of participants and inadequate consideration of confounding variables. These methodological limitations warrant improvement in future studies to confirm the findings in this review.

### Supplementary Information

**Supplementary Material 1.**

**Supplementary Material 2.**

**Supplementary Material 3.**

## Data Availability

All data generated or analysed during this study are included in this published article and its supplementary information files.

## Abbreviations

PRISMA: Preferred Reporting Items for Systematic reviews and Meta-Analyses
RoBANS: Risk of Bias Assessment Tool for Non-randomized Studies
BPSD: Behavioral and psychological symptoms of dementia
bvFTD: Behavioral variant of frontotemporal dementia
ADAD: Autosomal dominant Alzheimer’s disease
