# Supplementary material for: Similarities and differences in the prevalence and risk factors of suicidal behavior between caregivers and people with dementia: a systematic review
Source: BMC Geriatr. 2024 Mar 14;24:254. doi: 10.1186/s12877-024-04753-4 (PMC10941364; doi:10.1186/s12877-024-04753-4)
Supplement: Supplementary file 1 — Supplementary Material 1. [file 12877_2024_4753_MOESM1_ESM.docx]

**Additional file 1. List of journals where hand searching was performed**

| **No.** | **List of journals** |
| --- | --- |
| 1.  2.  3.  4.  5.  6.  7.  8.  9.  10.  11.  12.  13.  14.  15.  16.  17.  18.  19.  20.  21.  22.  23.  24.  25.  26.  27.  28.  29.  30.  31.  32.  33.  34.  35.  36  37.  38.  39.  40.  41.  42.  43.  44.  45.  46.  47.  48.  49.  50.  51.  52.  53. | Alzheimer’s and Dementia  Work, Aging and Retirement  Journal of the American Geriatrics Society  American Journal of Geriatric Psychiatry  GeroScience  Age and Ageing  Aging and Disease  The Gerontologist  Journals of Gerontology - Series B Psychological Sciences and Social Sciences  NPJ Aging and Mechanisms of Disease  Psychology and Aging  Gerontology  Journal of Alzheimer's Disease  BMC Geriatrics  Journal of Nutrition, Health and Aging  European Review of Aging and Physical Activity  International Journal of Geriatric Psychiatry  International Psychogeriatrics  Aging and Mental Health  Archives of Gerontology and Geriatrics  Journal of Aging and Health  JMIR Aging  Alzheimer Disease and Associated Disorders  Dementia and Geriatric Cognitive Disorders  European Journal of Ageing  Journal of Geriatric Psychiatry and Neurology  Geriatrics and Gerontology International  Canadian Geriatrics Journal  Clinical Gerontologist  European Geriatric Medicine  Annals of Geriatric Medicine and Research  Journal of Elder Abuse and Neglect  Australasian Journal on Ageing  American Journal of Alzheimer's Disease and other Dementias  Journal of Aging Research  Interdisciplinary Topics in Gerontology and Geriatrics  Geriatrics (Switzerland)  Current Gerontology and Geriatrics Research  Psychogeriatrics  Research in gerontological nursing  Dementia e Neuropsychologia  GeroPsych: The Journal of Gerontopsychology and Geriatric Psychiatry  Gerontology and Geriatrics Education  Current Geriatrics Reports  Gerontology and Geriatric Medicine  International Journal of Gerontology  Journal of Alzheimer's Disease Reports  Alzheimer's & Dementia : Diagnosis, Assessment & Disease Monitoring  Alzheimer's Research & Therapy  American Journal of Alzheimer's Disease & Other Dementias®  Dementia & Neuropsychologia  Dementia and Geriatric Cognitive Disorders Extra  Dementia and Neurocognitive Disorders |
