# Supplementary material for: Similarities and differences in the prevalence and risk factors of suicidal behavior between caregivers and people with dementia: a systematic review
Source: BMC Geriatr. 2024 Mar 14;24:254. doi: 10.1186/s12877-024-04753-4 (PMC10941364; doi:10.1186/s12877-024-04753-4)
Supplement: Supplementary file 2 — Supplementary Material 2. [file 12877_2024_4753_MOESM2_ESM.docx]

**Additional file 2: Detailed search steps performed on PubMed**

Electronic search of published literature from 1950 to 2023 was carried out on PubMed. Initially, a preliminary search was performed using Medical Subject Headings (MeSH) keywords such as “dementia” and “suicide”. Then, the search continued with more specific words such as “suicide and people with dementia”, “caregivers of people with dementia” and “suicide” OR “suicide and caregivers of people with dementia”.

Dual independent review of the search results was carried out by two authors (MFILBA and NIS). During the title and abstract screening stage, 1139 articles were obtained on suicide and dementia patients and 69 articles were obtained on suicide and caregivers of dementia patients. After screening abstracts for duplicate and eligibility criteria, a total of 15 articles on suicidal behavior among caregivers of dementia patients and a total of 85 articles on suicidal behavior among people with dementia remained. After full text screening, a final total of 8 articles on suicidal behavior among caregivers of dementia patients and 24 articles on suicidal behavior among people with dementia were selected after excluding 68 articles (due to their focus on assisted suicide; or suicide among patients of illnesses and caregivers of patients with illnesses other than dementia; or articles with insufficient information such as studies which did not include study type, participant recruitment and selection, study duration, diagnostic criteria, sociodemographics, missing data, estimation of effect with confidence intervals and p-value, or key conclusion). During screening for the inclusion of the articles for review, any discrepancies in the findings between the two authors were discussed and resolved. If there was difficulty to resolve the discrepancies, then the opinion of the 3rd author (MAM) was sought.
