# Supplementary material for: Similarities and differences in the prevalence and risk factors of suicidal behavior between caregivers and people with dementia: a systematic review
Source: BMC Geriatr. 2024 Mar 14;24:254. doi: 10.1186/s12877-024-04753-4 (PMC10941364; doi:10.1186/s12877-024-04753-4)
Supplement: Supplementary file 3 — Supplementary Material 3. [file 12877_2024_4753_MOESM3_ESM.docx]

**Additional File 3. Risk of bias assessment of the selected studies in this systematic review measured by the Risk of Bias Assessment Tool for Non-randomized Studies (RoBANS)**

| **Selected studies** | **Selection of participants** | **Confounding variables** | **Measurement of exposure** | **Blinding of outcome assessment** | **Incomplete outcome data** | **Selective outcome reporting** | **Overall risk of bias** |
| --- | --- | --- | --- | --- | --- | --- | --- |
| Hosaka and Sugiyama, 2003 | High risk | High risk | Low risk | Unclear risk | Low risk | Low risk | Unclear risk |
| Shaji et al., 2003 | High risk | High risk | High risk | Unclear risk | Low risk | Low risk | Unclear risk |
| Valente et al., 2011 | High risk | High risk | Low risk | Unclear risk | Low risk | Low risk | Unclear risk |
| O’Dwyer et al., 2012 | High risk | High risk | Low risk | Unclear risk | Low risk | Low risk | Unclear risk |
| O’Dwyer et al., 2013 | High risk | High risk | High risk | Unclear risk | Low risk | Low risk | Unclear risk |
| Lewis, 2015 | High risk | High risk | High risk | Unclear risk | Low risk | Low risk | Unclear risk |
| Koyama et al., 2016 | High risk | Low risk | Low risk | Unclear risk | Low risk | Low risk | Unclear risk |
| Joling et al., 2017 | Low risk | High risk | Low risk | Unclear risk | Low risk | Low risk | Unclear risk |
| Anderson et al., 2019 | High risk | High risk | High risk | Unclear risk | Low risk | Low risk | Unclear risk |
| Joling et al., 2019 | High risk | Low risk | Low risk | Unclear risk | Low risk | Low risk | Unclear risk |
| Kim et al., 2019 | High risk | High risk | High risk | Unclear risk | Low risk | Low risk | Unclear risk |
| Rosato et al., 2019 | High risk | High risk | Low risk | Unclear risk | Low risk | Low risk | Unclear risk |
| Lyness et al., 1992 | Low risk | High risk | Low risk | Unclear risk | Low risk | Low risk | Unclear risk |
| Florrio et al., 1997 | Low risk | High risk | Low risk | Unclear risk | Low risk | Low risk | Unclear risk |
| Rao et al., 1997 | Low risk | High risk | Low risk | Unclear risk | Low risk | Low risk | Unclear risk |
| Rubro et al., 2001 | High risk | Low risk | Low risk | Unclear risk | Low risk | Low risk | Unclear risk |
| Draper et al., 2003 | Low risk | High risk | Low risk | Unclear risk | Low risk | Low risk | Unclear risk |
| Heun et al., 2003 | High risk | Low risk | Low risk | Unclear risk | Low risk | Low risk | Unclear risk |
| Peisah et al., 2006 | High risk | High risk | Low risk | Unclear risk | Low risk | Low risk | Unclear risk |
| Erlangsen et al., 2008 | High risk | High risk | Low risk | Unclear risk | Low risk | Low risk | Unclear risk |
| Purandane et al., 2009 | Low risk | Low risk | Low risk | Unclear risk | Low risk | Low risk | Low risk |
| Qin, 2011 | Low risk | Low risk | Low risk | Unclear risk | Low risk | Low risk | Low risk |
| McCarthy et al., 2012 | Low risk | High risk | Low risk | Unclear risk | Low risk | Low risk | Unclear risk |
| Borges et al., 2014 | High risk | Low risk | Low risk | Unclear risk | Low risk | Low risk | Unclear risk |
| Randall et al., 2014 | Low risk | Unclear risk | Low risk | Unclear risk | Low risk | Low risk | Unclear risk |
| Nishida et al., 2015 | High risk | Unclear risk | Low risk | Unclear risk | Low risk | Low risk | Unclear risk |
| Matschke et al., 2018 | Low risk | Unclear risk | Low risk | Unclear risk | Low risk | Low risk | Unclear risk |
| Morgan et al., 2018 | High risk | Unclear risk | Low risk | Unclear risk | Low risk | Low risk | Unclear risk |
| Zucca et al., 2019 | High risk | High risk | Low risk | Unclear risk | Low risk | Low risk | Unclear risk |
| Ng et al., 2020 | High risk | High risk | Low risk | Unclear risk | Low risk | Low risk | Unclear risk |
| Ortner et al., 2021 | High risk | High risk | Low risk | Unclear risk | Low risk | Low risk | Unclear risk |
| Alothman et al., 2022 | Low risk | Unclear risk | Low risk | Unclear risk | Low risk | Low risk | Unclear risk |
| Barak et al., 2002 | High risk | High risk | Low risk | Unclear risk | Low risk | Low risk | Unclear risk |
| Seyfried et al., 2011 | High risk | High risk | Low risk | Unclear risk | Low risk | Low risk | Unclear risk |
| Tu et al., 2016 | Low risk | Unclear risk | Low risk | Unclear risk | Low risk | Low risk | Unclear risk |
| Annor et al., 2019 | High risk | High risk | Low risk | Unclear risk | Low risk | Low risk | Unclear risk |
| Choi et al., 2021 | Low risk | Low risk | Low risk | Unclear risk | Low risk | Low risk | Low risk |
| Gunak et al., 2021 | Low risk | Unclear risk | Low risk | Unclear risk | Low risk | Low risk | Unclear risk |
| Holmstrand et al., 2021 | High risk | Low risk | Unclear risk | Unclear risk | Low risk | Low risk | Unclear risk |
| Schmutte et al., 2022 | High risk | High risk | Low risk | Unclear risk | Low risk | Low risk | Unclear risk |
